# Minimally Invasive Versus Open Pyloromyotomy for Infantile Hypertrophic Pyloric Stenosis: Insights from an Updated Systematic Review and Meta-Analysis

**DOI:** 10.3390/pediatric17060124

**Published:** 2025-11-10

**Authors:** Amani N. Al-Ansari, Sagar Ahammed, Ahmed A. Sofy, Somaya Shokry Tawfik

**Affiliations:** 1 Department of Pediatric Surgery, Hamad Medical Corporation, Doha 3050, Qatar; 2 Department of Oncology, Khwaja Yunus Ali Medical College and Hospital, Enayetpur, Sirajganj 6751, Bangladesh; drsagarahammed@gmail.com; 3 Faculty of Medicine, Fayoum University, Fayoum 63514, Egypt; ahmedadel40222@gmail.com (A.A.S.); somayashokry8@gmail.com (S.S.T.)

**Keywords:** pyloric stenosis, laparoscopic pyloromyotomy, open pyloromyotomy, minimally

## Abstract

**Background:** Infantile hypertrophic pyloric stenosis represents one of the most prevalent gastrointestinal disorders in infants. It presents with severe persistent vomiting and electrolyte imbalance. Pyloromyotomy is the gold standard approach in the management of pyloric stenosis. The laparoscopic approach provides a reliable and safe alternative to the open technique. We aimed to compare the surgical outcomes of both approaches and determine which approach is superior to the other. **Methods:** We searched for relevant articles by searching Scopus, Web of Science, PubMed, and the Cochrane Library until January 2025. The Cochrane risk of bias tool was utilized to assess the quality of the clinical trials, whereas the ROBINS-I tool was used in the observational studies. Our primary outcomes were operation time, length of hospital stay, time needed for full feeding, incidence of incomplete pyloromyotomy, mucosal perforation, wound infection, postoperative vomiting, postoperative incisional hernia, postoperative seroma or hematoma formation, need for reoperation, and rate of conversion to P in the laparoscopic group. **Results:** We included 12 eligible articles that compared laparoscopic pyloromyotomy with open pyloromyotomy in infants with hypertrophic pyloric stenosis. Our analysis revealed comparable results for both procedures in terms of operation time (*p* = 0.83), hospitalization duration (*p* = 0.06), mucosal perforation (*p* = 0.49), postoperative complications such as vomiting (*p* = 0.10), incisional hernia (*p* = 0.60), seroma (*p* = 0.52), and reoperation rates (*p* = 0.17). Patients who underwent LP achieved full feeding in less time (*p* = 0.007) and had fewer wound infections (*p* = 0.01) compared to OP. However, the incidence of incomplete pyloromyotomy was lower in the OP group than in the LP group (*p* = 0.03). **Conclusions:** Both open and laparoscopic pyloromyotomy are effective for treating hypertrophic pyloric stenosis. The laparoscopic approach offers the advantages of a faster return to full feeding and lower wound infection rates but increases the risk of incomplete pyloromyotomy compared to the open technique. Surgeon preference and experience play crucial roles in surgical outcomes, provided that there is a thorough understanding of the benefits and limitations of both techniques.

## 1. Introduction

Infantile hypertrophic pyloric stenosis (IIHPS) is considered one of the most common gastrointestinal disorders occurring in infants, most commonly between the third and tenth weeks of age. However, later presentation have been reported [1,2,3]. IHPS occurs at a frequency of 2–4 per 1000 live births, with a male predominance [4,5]. Although there is no clear etiology of IHPS, it is well-documented that being firstborn and having preterm birth increases the risk of IHPS by 1.5-fold [6]. Previous studies reported a predominance of IHPS among White infants compared with Asian, Black, and Hispanic infants [7,8,9]. Additionally, there is an increased incidence of IHPS in newborns from industrial countries, including England, Northern Ireland, Scotland, and Wales [10]. IHPS arises from hyperplasia and hypertrophy of the pylorus, which represents the stomach outlet. This impairment of gastric emptying causes persistent vomiting, dehydration, electrolyte disturbance, and failure to thrive [11,12].

Pyloromyotomy involves a longitudinal incision and splitting of the seromuscular layers along the entire length of the hypertrophic pylorus muscles, which is the gold standard for managing children with IHPS [13,14]. Conventional pyloromyotomy is performed via either a supraumbilical incision or a right upper quadrant incision. A previous study demonstrated that, compared with laparoscopic pyloromyotomy (LP), open pyloromyotomy (OP) was associated with abdominal scarring and poor cosmetic results but had a similar complication rate [13]. LP is a minimally invasive procedure that has gained popularity since its first use in 1990 by Alain et al. [15]. The laparoscopic approach has various advantages, such as minimal tissue injury, better cosmesis, shorter postoperative hospitalization, shorter time to return to oral feed, and overall quicker recovery after surgery [16,17]. Several systematic reviews and meta-analyses have previously compared laparoscopic and open pyloromyotomy, generally reporting comparable outcomes between the two approaches, with some trends favoring laparoscopic pyloromyotomy in recovery parameters and open pyloromyotomy in myotomy completeness. However, these analyses were limited by smaller sample sizes and inclusion restricted mainly to randomized controlled trials.

The present review expands upon prior work by incorporating both randomized and observational studies published up to January 2025, yielding the largest pooled cohort to date. This broader inclusion enhances the generalizability of findings and enables evaluation of infrequent outcomes such as conversion rates and postoperative complications.

Currently, there is controversy and debate among pediatric surgeons about whether LP is superior to OP; thus, in this study, we aimed to assess the differences in perioperative outcomes and the complications of both procedures in infants with IHPS.

## 2. Methods

The PRISMA guidelines were followed while performing our study [18].

### 2.1. Search and Information Databases

We used the following search strategy to search for relevant articles until January 2025: (“Pyloric Stenosis” OR “pyloric stenoses” OR “Hypertrophic Pyloric Stenosis” OR “ pyloromyotom* OR “Pylorus Stenosis” OR “pyloric obstruction”) AND (Laparoscope OR Laparoscopic Surgery OR Laparoscopy OR laparoscop*). The PubMed, Scopus, Web of Science, and Cochrane Library were the main online databases used.

### 2.2. Selection Criteria and Eligibility Criteria

After removing duplicates via EndNote software X8.0.1, the selection of the remaining articles was carried out in two stages. The first stage involved screening titles and abstracts to find eligible studies. After that, we screened all full texts that were selected from the first stage on the basis of our eligibility criteria.

### 2.3. Study Population and Design

Our studied population were infants with IHPS. Our intervention was pyloromyotomy using the laparoscopic approach compared to pyloromyotomy using the open approach. The main assessed outcomes were operation time, length of hospital stay, time needed for full feeding, incidence of incomplete pyloromyotomy, mucosal perforation, wound infection, postoperative vomiting, postoperative incisional hernia, postoperative seroma or hematoma formation, need for reoperation, and rate of conversion to OP in the laparoscopic group. Eligible study designs included randomized controlled trials (RCTs) and observational studies, such as case–control studies and case series. Studies were excluded if they did not report the outcomes of interest or if they were review articles, conference abstracts, posters, non-indexed publications, or secondary research, including systematic reviews and meta-analyses.

### 2.4. Data Extraction

Data from the included studies were retrieved and plotted on an Excel sheet. Two authors independently conducted the data extraction for both baseline and main outcomes, and a third author addressed any disagreements. We extracted data on the general studies and patients’ characteristics, including country, study design, age, weight, pyloric channel length, number of patients with malnutrition, follow-up duration, and the open group utilized procedure. Moreover, we extracted data of our main outcomes, such as operation time (minutes), length of hospital stay (hours), time needed for full feeding (hours), incidence of incomplete pyloromyotomy, mucosal perforation, wound infection, postoperative vomiting, postoperative incisional hernia, postoperative seroma or hematoma formation, need for reoperation, and rate of conversion to OP in the laparoscopic group.

### 2.5. Quality Assessment

We employed the Cochrane risk of bias assessment tool for RCTs [19]. In contrast, the ROBINS-I tool was used to evaluate the risk of bias in observational studies [20]. We included all studies regardless of the risk of bias results.

### 2.6. Qualitative Synthesis

All studies were incorporated in the qualitative analysis, especially when outcomes such as conversion rates to open procedures lacked sufficient data for quantitative pooling. Quantitative analysis was applied only to outcomes with analyzable data. This was decided after data collection from the included studies.

### 2.7. Statistical Analysis

We analyzed the outcome data using RevMan software 5.4. Mean difference (MD) and 95% confidence interval (CI) were implemented to analyze continuous outcomes. The dichotomous outcomes are expressed as risk ratios (RRs) and 95% confidence intervals. The pooled estimate was considered statistically significant if the *p* value was <0.05. The heterogeneity among the data were evaluated via the chi-square test *p* value and the I^2^ value. The outcome was considered heterogeneous if I^2^ > 50% or *p* < 0.1 [21]. A sensitivity analysis was performed on the heterogeneous outcomes (operation time, duration of hospital stay, and time to full feed), excluding studies that heavily affected the overall pooled estimate. A fixed-effect model was used if the outcome was homogeneous. However, the random effects model was restricted to heterogeneous outcomes.

## 3. Results

### 3.1. Summary of the Included Studies

**Figure 1:**
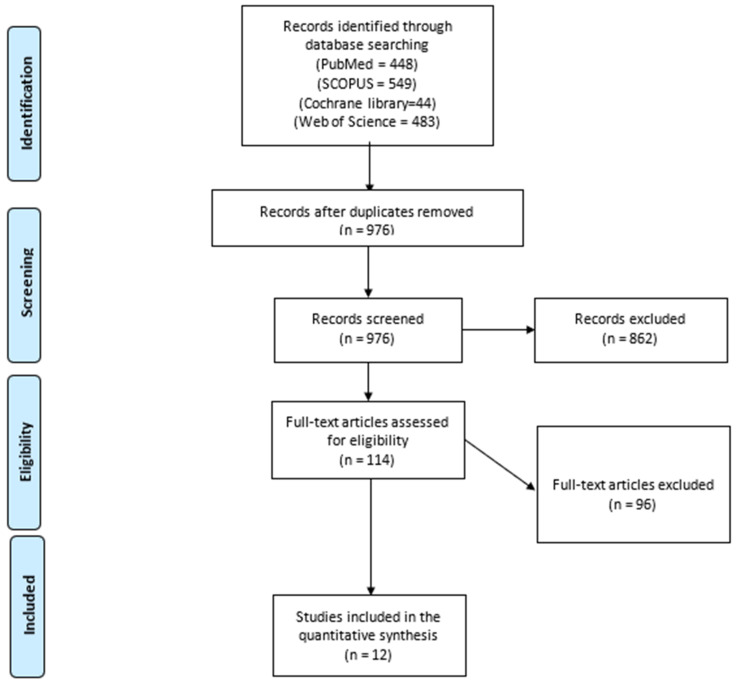
The PRISMA flow diagram.

We present our search process in the PRISMA flow diagram in Figure 1. A total of 676 records were screened after removing duplicates. A total of 114 studies were selected for eligible screening. We finally analyzed 1672 patients from 12 included studies [13,16,22,23,24,25,26,27,28,29,30,31] that compared LP with OP in infants with IHPS regarding the surgical outcomes and the safety of both techniques. We included nine RCTs and three observational studies. The baseline and demographic characteristics of the included studies and patients and the differences in age, gender, and special population are illustrated in Table 1.

### 3.2. Risk of Bias Assessment

**Figure 2:**
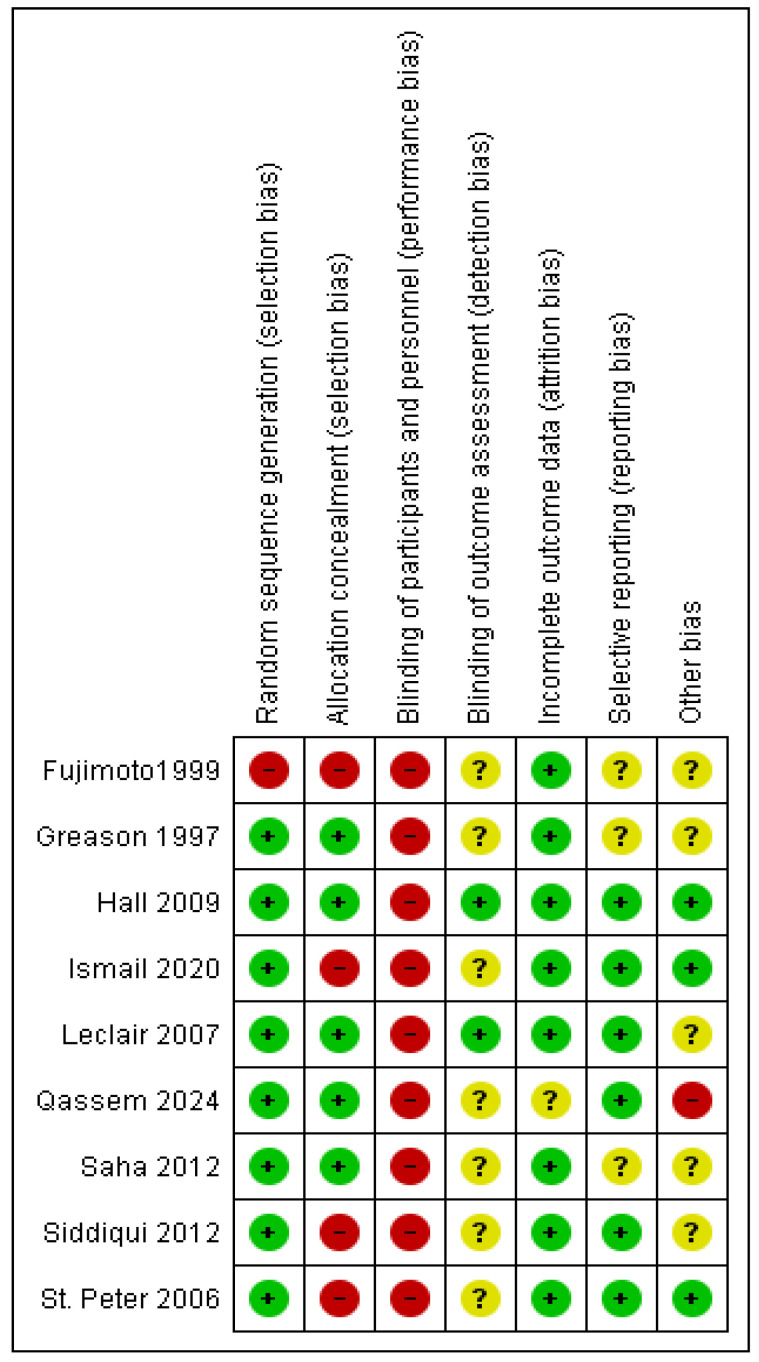
The risk of bias summary of the included trials [13,16,22,23,24,26,27,28,29].

The quality assessment of the included RCTs according to the Cochrane risk of bias tool revealed an overall moderate risk of bias, as shown in Figure 2. The assessment of the included observational studies revealed an overall moderate risk of bias according to the ROBINS-I risk of bias tool, as described in Table 2.

### 3.3. Analysis of Outcomes

#### 3.3.1. Operation Time (Min)

**Figure 3:**
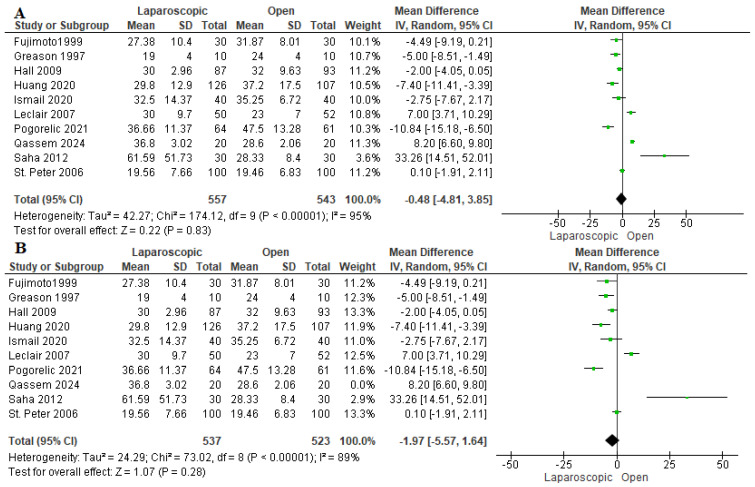
The forest plot of the operation time outcome. (**A**) represents the analysis before the sensitivity analysis. (**B**) represents the pooled analysis after sensitivity analysis [16,22,23,24,26,27,28,29,30,31].

The operation time was reported in ten studies assessing 1100 patients [16,22,23,24,26,27,28,29,30,31]. The pooled estimate revealed nonsignificant variation between the two techniques (mean difference = 0.48 min [−4.81, 3.85 min], (*p* = 0.83). We observed heterogeneity among the studies (I^2^ = 95%) (Figure 3B). Thus, we performed a sensitivity analysis excluding Qassem et al. 2024 [28]. After sensitivity analysis, we also found no difference between both cohorts (mean difference −1.97 min [−5.57, 1.64] (I^2^ = 89%)) (Figure 3B). Excluding Qassem et al. decreased the overall heterogeneity as they included the least weights among infants in our study, which may be associated with longer operation time compared to the rest of the studies [32].

#### 3.3.2. Length of Hospital Stay (Hours)

**Figure 4:**
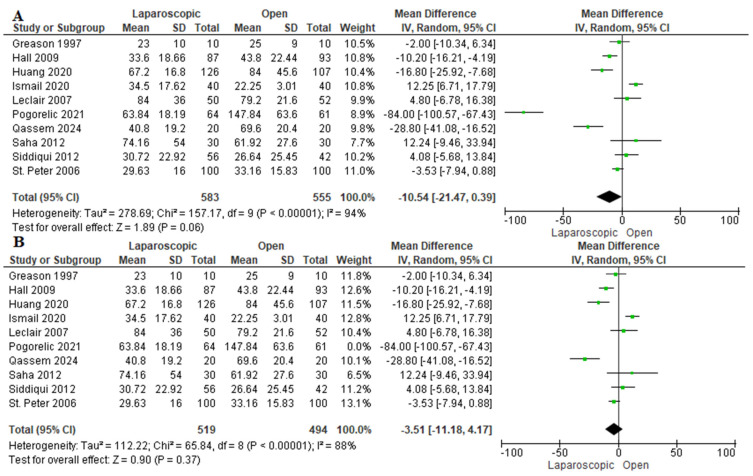
The forest plot of the length of hospital stay outcome. (**A**) represents the analysis before the sensitivity analysis. (**B**) represents the pooled analysis after sensitivity analysis [13,16,22,24,26,27,28,29,30,31].

Ten studies evaluated the duration of hospitalization [13,16,22,24,26,27,28,29,30,31]. We observed a nonsignificant shorter length of hospital stay among patients who underwent LP by −10.54 h [−21.47, 0.39 h] (*p* = 0.06). The data were heterogeneous (*p* < 0. 001); I^2^ = 94% (Figure 4A). A sensitivity analysis was performed, excluding Pogorelić et al., 2021 [30]. After sensitivity analysis, we also found a similar length of hospital stay (mean difference −3.51 h [−11.18, 4.17 h], I^2^ = 88%) (Figure 4B). Pogorelić et al., 2021 [30] reported that patients allocated to the open group required a much longer duration of hospital stay compared to the laparoscopic group and the open groups in the other included studies, which may be explained by a higher incidence of postoperative complications among patients in the open group. Furthermore, they did not consider the experience of the surgeon in their study, which may greatly impact the incidence of complications and postoperative hospital stay.

#### 3.3.3. Time to Full Feeding (Hours)

**Figure 5:**
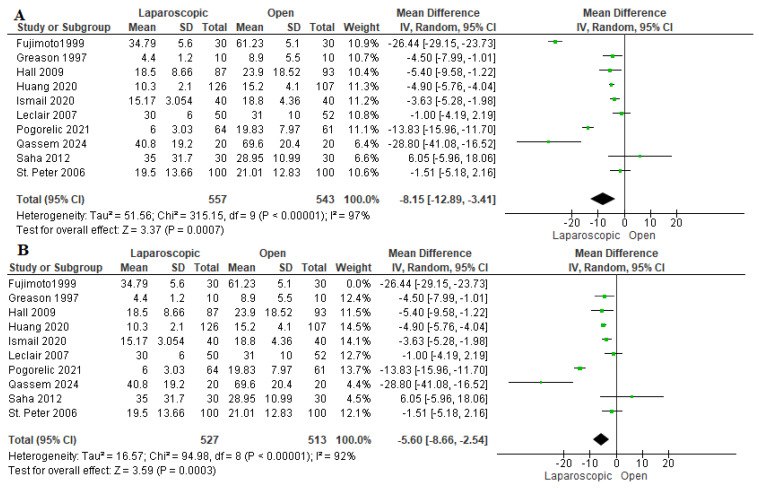
The forest plot of the time to full feeding outcome. (**A**) represents the analysis before the sensitivity analysis. (**B**) represents the pooled analysis after sensitivity analysis [16,22,23,24,26,27,28,29,30,31].

The time needed to achieve full feeding was assessed in 10 studies, including 1100 patients [16,22,23,24,26,27,28,29,30,31]. Pooled analysis revealed that patients who underwent LP needed significantly less time to achieve full feeding than those who were allocated to the OP group (mean difference −8.15 h [−12.89, −3.41 h], *p* = 0.007) (Figure 5A). Due to the considerable heterogeneity among studies (I^2^ = 97%), a sensitivity analysis in which Fujimoto et al. (1999) [23] was removed was performed, yielding an overall favoring of the LP group over the OP group (mean difference = −5.60 h [−8.66, −2.54 h], (I^2^ = 92%)) (Figure 5B). Fujimoto et al. (1999) [23] reported a significantly longer time needed to achieve full feeding compared to other studies. This may be due to the lack of experience of the senior registrar who performed both operations. Additionally, the laparoscopic approach was considered a recent approach which was harder than the usual open approach, which is why most pediatric surgeons preferred the conventional approach for performing pyloromyotomy [23,26,33].

#### 3.3.4. Incomplete Pyloromyotomy

**Figure 6:**
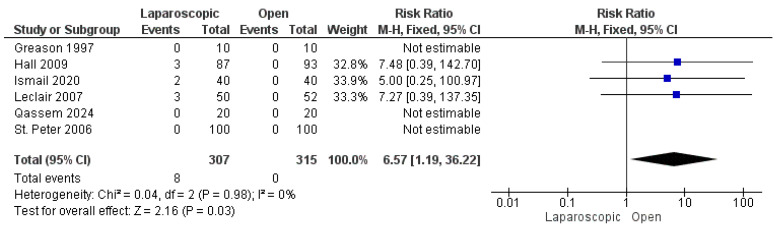
The forest plot of the incomplete pyloromyotomy outcome [16,22,24,26,27,28].

Six studies reported the incidence of incomplete pyloromyotomy [16,22,24,26,27,28]. A total of 8 patients out of 622 experienced incomplete pyloromyotomy, all in the LP group, with a significant favoring of the OP technique over the LP technique (risk ratio = 6.57 [1.19, 36.22], (*p* = 0.03). The pooled analysis was homogeneous (*p* = 0.98); I^2^ = 0% (Figure 6).

#### 3.3.5. Mucosal Perforation

**Figure 7:**
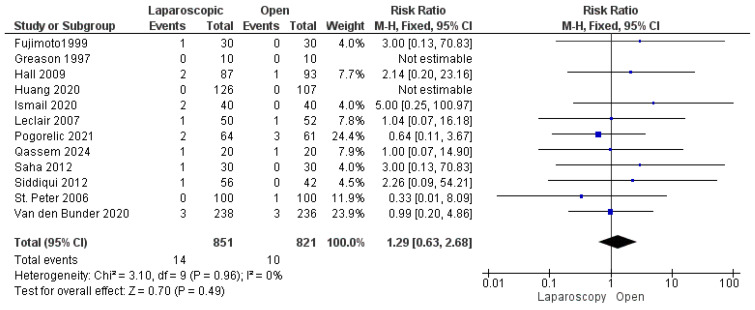
The forest plot of the mucosal perforation outcome [13,16,22,23,24,25,26,27,28,29,30,31].

The risk of mucosal perforation was reported by all included studies [13,16,22,23,24,25,26,27,28,29,30,31], analyzing 1672 patients. The overall risk ratio revealed a nonsignificant greater risk of mucosal perforation in the LP group than in the OP group (risk ratio = 1.29 [0.63, 2.68], (*p* = 0.49). Homogeneity was observed among the studies (*p* = 0.96); I^2^ = 0% (Figure 7).

#### 3.3.6. Wound Infection

**Figure 8:**
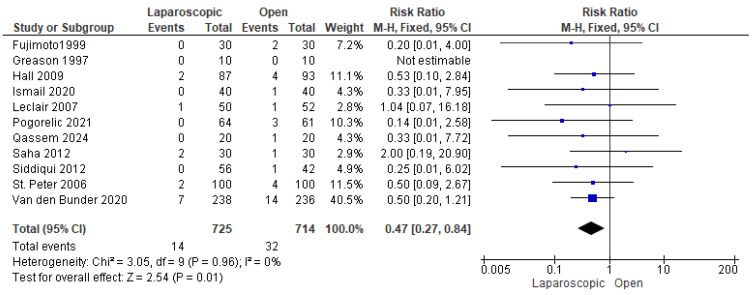
The forest plot of the wound infection outcome [13,16,22,23,24,25,26,27,28,29,30].

Eleven studies reported the incidence of wound infection among patients in both groups [13,16,22,23,24,25,26,27,28,29,30]. Wound infection was observed in 46 patients out of 1439 (3.19%), 14 in the LP group and 32 in the OP group. The combined risk ratio revealed a significantly lower risk of wound infection in the LP group compared to the OP group (risk ratio = 0.47 [0.27, 0.84], (*p* = 0.01). No heterogeneity was detected among the data (*p* = 0.96); I^2^ = 0% (Figure 8).

#### 3.3.7. Postoperative Vomiting

**Figure 9:**
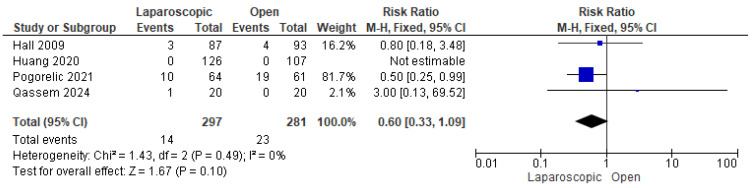
The forest plot of the postoperative vomiting outcome [24,28,30,31].

Four studies [24,28,30,31] assessed postoperative vomiting. A total of 37 out of 578 patients experienced postoperative vomiting. The combined analysis showed a nonsignificant lower risk of postoperative vomiting among children who underwent LP compared with OP (risk ratio = 0.60 [0.33, 1.09], (*p* = 0.10)). The data were homogeneous (*p* = 0.49); I^2^ = 0% (Figure 9).

#### 3.3.8. Postoperative Incisional Hernia

**Figure 10:**
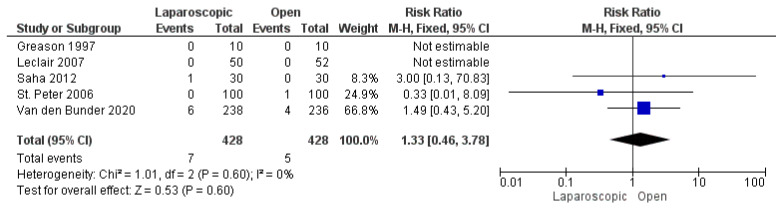
The forest plot of the postoperative incisional hernia outcome [16,25,26,27,29].

Five studies, including 856 patients, reported postoperative incisional hernia [16,25,26,27,29]. The overall risk ratio showed a comparable risk of postoperative incisional hernia in both techniques (risk ratio = 1.33 [0.46, 3.78], (*p* = 0.60)). The pooled analysis demonstrated homogeneity (*p* = 0.60); I^2^ = 0% (Figure 10).

#### 3.3.9. Postoperative Seroma or Hematoma Formation

**Figure 11:**
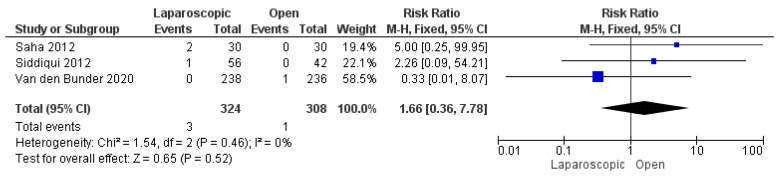
The forest plot of the postoperative seroma or hematoma formation outcome [13,25,29].

We analyzed 632 patients from three included studies [13,25,29]. The combined risk ratio was not significantly different between the two groups (risk ratio = 1.66 [0.36, 7.78], (*p* = 0.52)). We found no inconsistency among the studies (*p* = 0.46); I^2^ = 0% (Figure 11).

#### 3.3.10. Need for Reoperation

**Figure 12:**
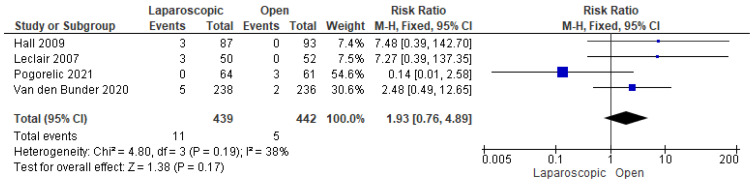
The forest plot of the need for reoperation outcome [16,24,25,30].

We demonstrated a total of 16 reoperations in 881 patients from four studies that reported this outcome [16,24,25,30]. Our analysis showed a nonsignificant higher risk of reoperation among patients who underwent LP (risk ratio = 1.93 [0.76, 4.89], (*p* = 0.17)). Data were consistent (*p* = 0.19); I^2^ = 38% (Figure 12).

#### 3.3.11. Conversion to OP

The incidence of conversion from LP to OP was assessed in 11 studies [13,16,22,23,24,25,26,27,28,29,31]. We demonstrated that a total of six conversions occurred in 787 patients (0.76%). Table 3 shows the rate of conversion from LP to OP among the studies.

## 4. Discussion

Pyloromyotomy is a highly effective modality for managing IHPS [14]. The widespread adoption of LP, a minimally invasive surgical technique, has established it as a safe and reliable alternative to OP in the surgical treatment of IHPS. The utilization of LP in the USA has significantly increased from 59% in 2013 to 65.5% in 2015 [34]. To date, conflicting evidence exists regarding the superiority of LP over OP in the surgical management of infants with IHPS, highlighting the need for further research to determine the optimal approach [22,35]. This disagreement may be due to surgeon preference, the higher costs and availability of specialized equipment, and the more experience required in laparoscopy than the traditional open surgery [34,35].

In our systematic review and meta-analysis, we compared LP and OP in infants with IHPS in terms of the surgical outcomes, safety, and efficacy of both techniques. Our analysis of 1672 patients revealed comparable outcomes between LP and OP in terms of operation time, hospitalization duration, mucosal perforation, and postoperative complications such as vomiting, incisional hernia, seroma, and reoperation rates. Patients who underwent LP achieved full feeding in less time and had fewer wound infections compared to OP. However, the incidence of incomplete pyloromyotomy was lower in the OP group than in the LP group.

In 2022, a Cochrane review by Staerkle et al. [36] assessed 720 patients from seven included studies and found that both techniques had similar surgical results and perioperative complications, with a small nonsignificant increase in mucosal perforation in the LP group and an increased incidence of incomplete pyloromyotomy in the OP group. This is in line with most of our findings. However, in our study, LP was more effective at minimizing wound infections and accelerating the time to full feeding. Furthermore, our analysis revealed eight cases of incomplete pyloromyotomy, all within the LP group, suggesting a greater reliability of OP for complete pyloromyotomy. Additionally, our study included a greater number of studies encompassing both randomized and observational studies, which allowed better assessment of the surgical outcomes.

This may be explained by including more recent studies in our meta-analysis with more expertise in such techniques. A previous study analyzing the LP learning curve revealed that the complication rate decreases by 30–40% after 70 operations. Additionally, the diminished tactile sensation and cautious approach to prevent mucosal perforation in the laparoscopic technique may explain the higher rate of incomplete pyloromyotomies [37]. To ensure proper myotomy, a previous retrospective study by Ostlie et al. [38] evaluated the optimum pyloromyotomy length. They reported that a 2 cm pyloromyotomy incision significantly reduces the incidence of incomplete pyloromyotomy. St. Peter et al. reported their adherence to the 2 cm incision rule, showing complete pyloromyotomies among all patients in the LP cohort [27]. Four included studies [16,24,25,30] reported the need for reoperation for various reasons. Ten out of sixteen reoperations were due to incomplete pyloromyotomies (62.5%). Pogorelić et al. [30] reported three reoperations due to diffuse peritonitis caused by mucosal perforation among patients in the OP group. Finally, Van den Bunder et al. [25] reported three reoperations due to overlooked mucosal perforation in two patients, and one patient underwent reoperation due to active bleeding with hemoglobin drop.

In 2022, He et al. performed a more recent meta-analysis of 680 infants, studying the safety and efficacy of OP versus LP in IHPS [39]. They reported that both techniques have similar postoperative complication rates. However, their analysis favored the LP technique over the OP technique with respect to the time needed to reach full feeding and the length of hospital stay. In comparison, the OP group was associated with a significantly lower incidence of incomplete pyloromyotomies, which is consistent with our findings. Costanzo et al. (2018) analyzed 3256 patients with IHPS and found lower morbidity in the LP group compared to the open technique (*p* = 0.007) [40]. These findings align with Kethman et al. [34], who reported fewer complications and shorter hospital stays with LP without any significant differences in readmissions or reoperations.

The higher incidence of incomplete pyloromyotomy found in the laparoscopic group may be attributed to many factors. First, the laparoscopic technique involves a distinct learning curve, requiring precise hand–eye coordination and limited tactile feedback, which can increase the likelihood of incomplete muscle division during the early phase of surgical experience. Second, inherent technical constraints of laparoscopy such as reduced depth perception and instrument angulation may further contribute to this complication [41].

Ismail et al. [22] and Leclair et al. [16] both reported lower postoperative pain scores and reduced analgesic requirements in patients who underwent LP than in those who underwent OP. This is due to the minimally invasive nature of LP, which causes less tissue trauma, resulting in reduced postoperative pain. Hall et al. (2009) [24] reported that patients who underwent laparoscopy required less analgesia. However, surprisingly, their analysis showed no notable difference in pain scores between LP and OP [19]. Several studies have shown that the laparoscopic technique yields superior cosmetic results, with higher patient satisfaction and improved body image scores compared to OP [13,22,28].

Our study represents the most recent and largest-scale meta-analysis comparing LP with OP in infants with IHPS. We acknowledge various limitations in our study. First, the heterogeneity observed in certain outcomes may be attributed to variations in study designs across the included studies. Second, differences in institutional settings and the varying levels of surgical expertise among surgeons could have influenced the results. Third, the study does not account for the laparoscopic experience of individual surgeons, which may impact surgical outcomes. Fourth, it was not possible to distinguish between different open surgical approaches, such as the traditional right upper quadrant transverse incision and the transumbilical incision, which may influence operative time and cosmetic outcomes, Lastly, variability in postoperative refeeding protocols among studies may have contributed to the observed heterogeneity.

## 5. Conclusions

We believe that both OP and LP are reliable and safe options for managing IHPS, with the superiority of LP in terms of time to full feeding and the incidence of wound infection. However, LP is associated with a higher incidence of incomplete pyloromyotomy. Surgeon preference and experience play crucial roles in surgical outcomes, provided that there is a thorough understanding of the benefits and limitations of both techniques. LP may be preferred when surgical expertise is available, while surgery remains safer in low-resource or inexperienced settings. To better determine the factors influencing surgical success and assess the impact of surgeon expertise and preference on outcomes, further high-quality clinical trials with stronger evidence are needed.

## Figures and Tables

**Table 1:** Shows the baseline and demographic characteristics of the included studies and participants.

Study	Study Design	Country	Sample Size		Males (%)		Age (Days)		Weight (Grams)		Pyloric Channel Length (mm)		Malnutrition		Follow-Up	Open Group Surgical Procedure
			LP	OP	LP	OP	LP	OP	LP	OP	LP	OP	LP	OP		
**Greason 1997** [26]	RCT	USA	10	10	NR		34 ± 15	31 ± 18	4.1 ± 0.6	4.1 ± 0.7	NR	NR	NR	NR	NR	supra-umbilical incision
**Fujimoto 1999** [23]	RCT	Japan	30	30	NR		44.23 ± 15.74	43.63 ± 18.85	4061 ± 604	3805 ± 5611	25	23	NR	NR	NR	supra-umbilical incision
**St. Peter 2006** [27]	RCT	USA	100	100	NR		37.31 ± 1.47	36.68 ± 1.75	NR	NR	19.4	19.5	NR	NR	2 weeks	upper right transverse incision
**Leclair 2007** [16]	RCT	France	50	52	40 (80)	45 (87)	39 ± 17	35 ± 13	3780 ± 650	3790 ± 510	20.3	20.1	NR	NR	4–9 weeks	supra-umbilical incision
**Hall 2009** [24]	RCT	UK	87	93	NR		33.66 ± 11.3	34.33 ± 12	3800 ± 678.4	3800 ± 602.4	NR	NR	NR	NR	39 days	supra-umbilical incision
**Saha 2012** [29]	RCT	Bangladesh	30	30	NR		41.3	43.2	NR	NR	NR	NR	NR	NR	3 months	upper right transverse incision
**Siddiqui 2012** [13]	RCT	USA	56	42	41 (73)	33 (78)	36	37	4000	3800	NR	NR	NR	NR	56 months	upper right transverse incision
**Van den Bunder 2020** [25]	Retrospective study	Netherlands	238	236	401 (85)		35	32.5	NR	NR	NR	NR	NR	NR	2–3 weeks	supra-umbilical incision
**Huang 2020** [31]	Retrospective study	China	126	107	95 (75)	81 (75)	41.4 ± 10.8	38.8 ± 12.1	4300 ± 1300	4100 ± 1500	NR	NR	26 (20.6%)	21 (19.6%)	1 year	upper right transverse incision
**Ismail 2020** [22]	RCT	Egypt	40	40	NR		NR	NR	NR	NR	NR	NR	NR	NR	6 months	upper right transverse incision
**Pogoreli’c 2021** [30]	Retrospective study	Croatia	64	61	54 (85)	50 (82)	31 ± 10.6	34.53 ± 17	3618.3 ± 754.62	3821.66 ± 542.88	19.33 ± 2.27	19.5 ± 2.65	11 (17.8%)	16 (26.2%)	30 days	upper right transverse incision
**Qassem 2024** [28]	RCT	Egypt	20	20	31 (77.5%)		32.9 ± 10.78		2970 ± 420		17.02 ± 2.09		NR	NR	NR	upper right transverse incision

Data are presented as mean± standard deviation, number (percentage). LP = laparoscopic pyloromyotomy, OP = open pyloromyotomy, RCT = randomized controlled trial, NR = not reported.

**Table 2:** The risk of bias assessment of the observational studies according to ROBINS-I.

Study	Bias Due to Confounding	Selection Bias	Bias in Classification of Interventions	Bias Due to Deviations from Intended Intervention	Bias Due to Missing Data	Bias in Measurement of Outcomes	Bias in Selection of Reported Result
**Huang 2020 [31]**	low	low	low	low	no information	no information	low
**Van den ** **Bunder 2020** [25]	low	low	low	low	no information	no information	low
**Pogorelic’ 2021 [30]**	low	low	moderate	moderate	low	no information	low

**Table 3:** Conversion rate from laparoscopic pyloromyotomy to open pyloromyotomy.

Study	The Rate of Conversion
Van den Bunder 2020 [25]	0% (0/238)
Fujimoto 1999 [23]	3.3% (1/30)
Greason 1997 [26]	0% (0/10)
Hall 2009 [24]	0% (0/87)
Huang 2020 [31]	0% (0/126)
Ismail 2020 [22]	5% (2/40)
Leclair 2007 [16]	0% (0/50)
St. Peter 2006 [27]	1% (1/100)
Qassem 2024 [28]	5% (1/20)
Saha 2012 [29]	0% (0/30)
Siddiqui 2012 [13]	1.78% (1/56)

## Data Availability

The original contributions presented in this study are included in the article. Further inquiries can be directed to the corresponding author.
